# Caring for individuals with a difference of sex development (DSD): a Consensus Statement

**DOI:** 10.1038/s41574-018-0010-8

**Published:** 2018-05-16

**Authors:** Martine Cools, Anna Nordenström, Ralitsa Robeva, Joanne Hall, Puck Westerveld, Christa Flück, Birgit Köhler, Marta Berra, Alexander Springer, Katinka Schweizer, Vickie Pasterski, on behalf of the COST Action BM1303 working group 1

**Affiliations:** 1 Department of Paediatric Endocrinology, Ghent University Hospital, University of Ghent, Ghent, Belgium; 2 Department of Women’s and Children’s Health, Paediatric Endocrinology Unit, Karolinska Institutet, Karolinska University Hospital, Stockholm, Sweden; 3 Clinical Center of Endocrinology and Gerontology, Medical University-Sofia, Medical Faculty, Sofia, Bulgaria; 4 CAH support group, Chester, UK; 5 DSDNederland, Amsterdam, Netherlands; 6 Paediatric Endocrinology and Diabetology, Department of Paediatrics and Department of Clinical Research, Inselspital, Bern University Hospital, University of Bern, Bern, Switzerland; 7 Department of Paediatric Endocrinology, Charité University Medicine, Humboldt University Berlin, Berlin, Germany; 8 Department of Obstetrics and Gynaecology, Ramazzini Hospital, AUSL Modena, Modena, Italy; 9 Department of Paediatric Surgery, Medical University Vienna, Vienna, Austria; 10 Institute for Sex Research and Forensic Psychiatry, University Clinic Hamburg-Eppendorf, Hamburg, Germany; 11 Department of Psychology, University of Cambridge, Cambridge, UK

**Keywords:** Gonadal disorders, Endocrine reproductive disorders, Public health, Diagnosis

## Abstract

The term differences of sex development (DSDs; also known as disorders of sex development) refers to a heterogeneous group of congenital conditions affecting human sex determination and differentiation. Several reports highlighting suboptimal physical and psychosexual outcomes in individuals who have a DSD led to a radical revision of nomenclature and management a decade ago. Whereas the resulting recommendations for holistic, multidisciplinary care seem to have been implemented rapidly in specialized paediatric services around the world, adolescents often experience difficulties in finding access to expert adult care and gradually or abruptly cease medical follow-up. Many adults with a DSD have health-related questions that remain unanswered owing to a lack of evidence pertaining to the natural evolution of the various conditions in later life stages. This Consensus Statement, developed by a European multidisciplinary group of experts, including patient representatives, summarizes evidence-based and experience-based recommendations for lifelong care and data collection in individuals with a DSD across ages and highlights clinical research priorities. By doing so, we hope to contribute to improving understanding and management of these conditions by involved medical professionals. In addition, we hope to give impetus to multicentre studies that will shed light on outcomes and comorbidities of DSD conditions across the lifespan.

## Abstract (web summary)

Differences of sex development (DSDs) comprise a large group of congenital conditions of the urogenital tract and reproductive system. This Consensus Statement summarizes recommendations for clinical indications, diagnosis and management of patients with a DSD.

## Introduction

**Fig. 1:**
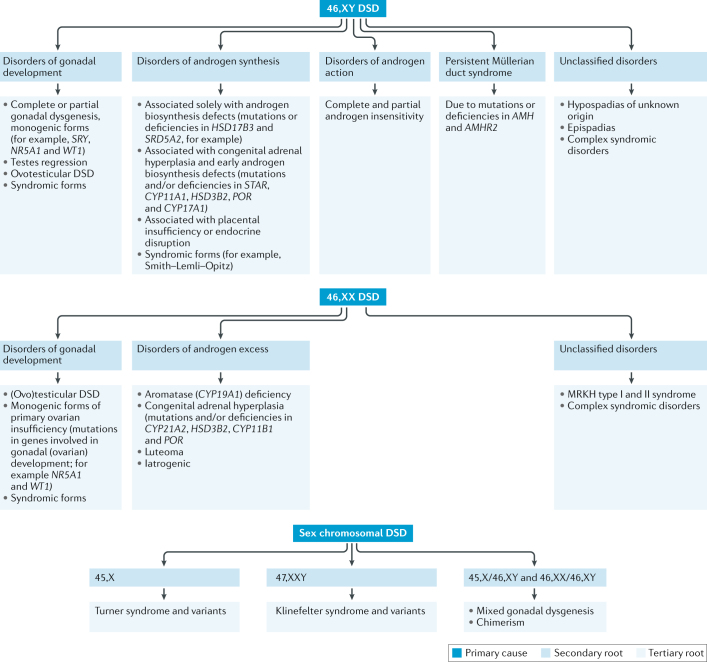
Classification of DSDs. Disorders of sex development (DSDs) are classified into three main groups on the basis of the karyotype of the affected individual (primary cause). Each main group encompasses several subgroups (secondary root) that orient towards a specific diagnosis (tertiary root). MRKH, Mayer–Rokitansky–Küster–Hauser syndrome.

Differences of sex development (DSDs; also known as disorders of sex development) comprise a large group of congenital conditions of the urogenital tract and reproductive system, affecting human sex determination and/or differentiation. Nomenclature remains controversial; current medical classification is largely based on the genetic status of the patient^1^ (Fig. 1). Our knowledge of DSDs has greatly evolved in the past decade owing to cutting-edge research on mammalian sex development and the genetic mechanisms underlying DSDs^2–4^. In parallel to this research, several clinical outcome studies have been conducted; however, the results of these studies are inconclusive owing to small and heterogeneous samples, variable methodology and the inclusion of older, sometimes outdated, treatments and surgical techniques. The lack of conclusive outcome data for patients with DSDs has triggered large-scale collaborative research that initially focused on basic science projects (such as the EuroDSD project) and since 2012 began addressing clinically oriented issues and outcomes (for example, dsd-LIFE). These clinically oriented studies have provided new insight on historical trends^5^, comorbidities^6^ and outcomes of specific conditions^7^. The development of international registries, such as the I-DSD Registry, the I-CAH Registry and the DSD Translational Research Network^8,9^, was crucial in the development of collaborative research projects. Now that the registries are established, however, emerging challenges include establishing ways for patients to access their personal data and developing methods for the optimal securement of privacy.

Recommendations from 2014, 2015 and 2016 address diagnostic strategies and provide guidance for a holistic clinical approach, mostly during childhood and adolescence^1,10,11^. Holistic approaches to treatment require multidisciplinary care by teams of relevant subspecialists working in close collaboration and a strong peer support network. Primary subspecialties include endocrinology, urology, gynaecology, andrology, psychology, nursing, social work, genetics and medical ethics. Input from support and service user groups has been paramount in defining new research questions. As a result, clinical studies focusing on comorbidities, treatment effects, outcomes in adults and psychosocial issues are placed high on the research agenda. How patients experience having a DSD and how they cope with medical procedures need to receive more attention in research and clinical practice^12^.

Indeed, insufficient, or inadequate, medical care might result in adverse patient outcomes that, in turn, could impede participation in studies aimed at further improving clinical care. For example, health-care systems that are unable to provide longitudinal holistic care, with coordinated transition to adult services, or that proceed with treatments with poor knowledge of the condition (for example, owing to lack of subspecialties) can cause mistrust and/or a lack of engagement with health-care professionals among adults with a DSD. Other factors that might result in adverse patient outcomes include non-acceptance of the chronic condition or avoidance of medical care due to negative experiences of medical procedures during childhood^13,14^. The rarity and complexity of the various conditions in combination with decentralization or lost follow-up of patients make studies including meaningful numbers of adults living with a DSD challenging. The resulting dearth of evidence-based guidelines for this cohort is then compounded further by demotivation of patients and caregivers to invest in sustainable health measures.

To address the aforementioned difficulties, we need to ensure that clinicians who do not have regular exposure to patients with a DSD have detailed guidance in clinical review and data collection for patients at various life stages. Ensuring this would increase the potential for meaningful results from clinical research and decrease the potential for bias in clinical assessments, thus facilitating longitudinal, multicentre studies as the foundation for evidence-based management and exchange of consistently formatted data in research networks such as I-DSD^15^. Finally, adherence to evidence-based protocols can serve as a quality indicator for defining centres of expertise. Many features and management issues are shared by the different DSDs. Most of the areas that are extensively discussed here mainly pertain to the 46,XY DSD, 46,XX DSD and 45,X/46,XY DSD groups (Fig. 1). Turner syndrome and Klinefelter syndrome, which are grouped under the wider DSD umbrella, require a similar multidisciplinary approach. For these conditions, however, clinical guidelines already exist and are widely used; therefore, they will not be addressed in this Consensus Statement^16–18^.

Standardizing the longitudinal assessment of individuals with a DSD across centres might also provide evidence for or against controversial procedures — such as surgical management of genitalia with an atypical appearance^19–21^ — and clinicians could then make decisions on the basis of careful consideration of relevant parameters. Parameters clinicians need to consider when deciding on the appropriate treatment strategy include body appearance, psychosocial support, sociocultural influences, gender-related development and genetic and/or biochemical background in addition to ethical, legal and human rights implications^1^. Intersex activists have encouraged legislative bodies worldwide to ban elective, irreversible genital surgery without the individual’s informed consent, and in some countries, such as Germany, legislative recommendations in this direction have been instituted^22^. In the absence of long-term outcome data that support or disfavour deferring genital surgery, there is currently little evidence that surgical practice has dramatically changed in recent years^20,23^. However, there is a broad consensus that alternatives to surgery, such as supporting families in parenting children who have a genital difference and/or facilitating psychological adjustment, have not been fully developed or supported by governmental health-care systems. Furthermore, there is a belief that stakeholders, most notably patient groups, have not been sufficiently consulted in this matter^24,25^. Finding ways to raise resilient children with atypical-appearing genitalia, defining the precise role of (early) genital surgery in the management of DSDs and collecting data on psychosocial adjustment and outcomes in both children who have undergone surgery and those who have not are considered top priorities.

From a patient perspective, structured and continued follow-up might increase beneficial interactions with health-care professionals, potentially enhancing understanding of the specific conditions, knowledge of future medical needs and compliance with the treatment^26^. A predefined schedule would greatly support patients and health-care staff during the vulnerable transition phase from adolescence to adulthood, encourage patients to prepare for discussions with caregivers and facilitate movement from one clinic to another by providing a personal summary of medical history^27–29^.

In this Consensus Statement, we aim to address the above outlined weaknesses in patient-centred care and research by reviewing existing evidence and, where this is absent, providing recommendations for holistic care and data collection in individuals with a DSD across ages. Definitions of several key terms used in this article are provided in Box 1.

Box 1 | Glossary of common terms **Chromatographic, mass spectrometric methods**

Tandem laboratory investigative techniques that can be used to analyse biochemical, organic and inorganic compounds commonly found in complex samples of environmental and biological origin.

**DSDs**

Congenital conditions in which chromosomal, gonadal or anatomical sex has been compromised, usually owing to a genetic mutation. Use of the term ‘disorder’ is controversial in the context of naturally occurring variations.

**Gender**

The psychological experience of being male, female, both or neither (typically used regarding social and/or cultural differences rather than biological ones).

**Gender identity**

The core sense of the gendered self as male, female or other.

**Gender role**

Behaviours, preferences and traits that differ, on average, between males and females in a given culture and historical period.

**Genome-wide sequencing**

A process used to determine the sequence of most of the DNA content (whole-genome sequencing) or most of the protein-encoding exons found in the genome (whole-exome sequencing) of an individual.

**Genotype**

The genetic make-up of an individual organism.

**Holistic health care**

A system of comprehensive patient care that considers the physical, emotional, social, economic and spiritual needs of the patient. Holistic care in DSDs also considers the needs of family members of patients.

**Intersex**

The term that was used prior to the 2006 Consensus Statement and that has been replaced by DSDs, mainly in the medical literature. Some individuals have re-appropriated the term and identify themselves as ‘intersex’, whether or not a DSD has been diagnosed.

**Phenotype**

The set of observable characteristics of an individual resulting from the interaction of its genotype with the environment.

**Sex**

The state of being male or female (typically used regarding biological differences that include sex chromosomes, gonads and internal and/or external reproductive structures).

**Sexual orientation**

The direction of sexual attractions to males, females, both or neither.

DSDs, differences of sex development (also known as disorders of sex development).

## Methods

This Consensus Statement was created within the framework of the European Cooperation in Science and Technology (COST) Action ‘DSDnet’, representing 23 European Union member states, 3 near-neighbouring countries and 5 international partner countries. The recommendations were developed following work around the theme ‘standardization of clinical assessment of individuals who have a DSD’ by a focus group of clinicians from relevant disciplines and patient representatives. Data were identified by searches of the PubMed and Embase databases and reference lists from relevant papers; only original research papers and reviews later than 2000 and published in English were considered. All data were subject to extensive group discussions, taking place at face-to-face meetings, until consensus on actual status summary and recommended actions was reached. The resulting document was posted on a members-only area of the DSDnet website in August 2017 for a vetting process by all stakeholders, including clinicians, scientists, ethicists, advocates and patient and parent representatives. General approval of the final Consensus Document was reached in September 2017.

## Individualized care plan

### Informed consent

Before undertaking any diagnostic and/or therapeutic procedure, patients, or parents and/or guardians of minors, need to be thoroughly informed by the clinical team and give consent. For sensitive and/or irreversible procedures, such as genital surgery, we advise that the intervention be postponed until the individual is old enough to be actively involved in the decision whenever possible. Informed patient and/or parental consent is also a prerequisite to collect data and biomaterials for research purposes; examples of such informed consent documents in multiple languages can be found in the I-DSD Registry. A discrepancy is often perceived between research topics proposed by scientists and what individuals who have a DSD consider important. In addition, many patients have felt abused in past research projects or mistreated by health professionals^12,30^. In response to these problems, collaborative networks, such as the DSDnet and ‘dsd-LIFE’ consortia, have been formed between researchers, health professionals, patient families and support groups. The dsd-LIFE study identified the research needs that patients and parents consider a priority (Box 2). In addition, in the newly established European Reference Network (ERN) covering rare endocrine conditions (Endo-ERN), patient participation in research and database management is considered crucial. Collaborations between patients, parents, health-care professionals and researchers are based on trust and confidentiality and must be handled with utmost care.

Box 2 Research priorities for patients and parents

- Information management
- Parental support
- Treatment choices and effects on health
- Comorbidities and care through the lifespan
- Tumour risk
- Outcomes in children and adults

### Diagnostic investigations

All individuals with a suspected DSD need a thorough diagnostic evaluation, including an extensive whole-body and genital physical exam, biochemical and genetic investigations and imaging studies, with results discussed in the multidisciplinary team^10^. The ultimate goal is to obtain a diagnosis at the molecular genetics level to allow prognostic predictions and genetic counselling and to set up an individualized management plan. Although the advent of genome-wide sequencing technologies has improved diagnostics, this goal cannot be achieved in ~50% of individuals with a 46,XY DSD, which highlights the importance of hitherto unidentified regulatory mechanisms^11,31,32^. Opportunities for advanced genetic work-up are currently mostly available through collaborative research projects that recruit patients enrolled in registries and networks; hence, participation of health-care providers in such networks is of direct benefit for patients. Biochemical work-up is equally important as it can, even in the absence of a genetic diagnosis, inform underlying pathology and hormonal treatments. Currently, chromatographic, mass spectrometric methods are recommended for exact steroid hormone measurements. However, these methods are not yet widely available, and standardization between laboratories has only just started^33^. As the diagnostic quest can be long and hard to bear for affected families, such uncertainty should not result in hesitation towards outlining the management plan and addressing pending clinical issues (for example, those regarding sex assignment and information sharing).

### Information and psychological support

Patients and parents are often facing a complex and previously unknown set of circumstances when they first learn about a DSD diagnosis. In addition, as part of holistic care, after the provision of medical information and clinical guidance patients and families should stay in regular contact with the clinical team (for example, once every 1–2 years) even in the absence of medical problems or treatments. Initial emotions can be overwhelming, and continued psychosocial support is often needed when families are processing the diagnosis as well as for further decision-making^34^. Such counselling might also provide support for communication within a patient’s social network and for the development of a personalized diagnosis-specific vocabulary, in addition to providing help for dealing with their feelings. Patient-centred support focuses on individual determinants of coping, such as personal attitudes, life experiences, cultural and religious background and eventual socio-economic barriers; however, it should also address fears, concerns and questions that are relevant for individuals and families across all DSDs (for example, how parents and/or guardians can inform their child about their DSD and how they can deal with negative reactions). If the families and patients have written information on these issues available at all times, it might further help them gain self-confidence to deal with the situation.

Tension exists with regards to the use of the terms ‘DSD’ versus ‘intersex’. Explaining the condition as a natural variation and promoting a non-binary view on sex and gender in general might alleviate this friction and direct the focus of discussion towards the provision of optimal care. If identified, most individuals prefer to use the specific name for their condition rather than the broader DSD or intersex terms^25^.

**Fig. 2:**
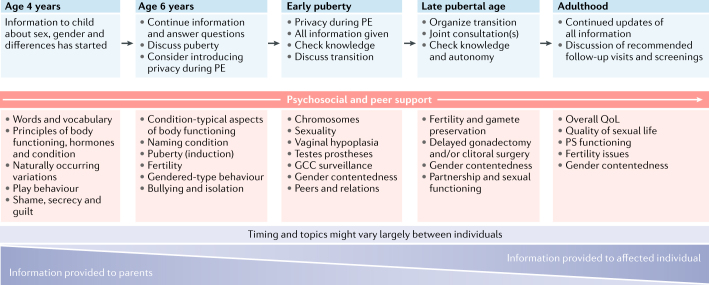
Multidisciplinary care and data collection in DSDs across ages. Multidisciplinary care and data collection begins at diagnosis and continues across the lifespan of the individual. The focus of the information process gradually shifts from parents to the affected child. Psychological and peer support are key elements at all ages. Although timing and topics may vary according to individual circumstances, it is generally agreed that children should be informed about their condition at an early age. Suggested themes to be discussed by team members are shown in blue boxes at the top of the figure, and (non-exhaustive) lists of important topics within these themes are represented in pink boxes in the centre of the figure. DSDs, differences of sex development; GCC, germ cell cancer; PE, physical examination; PS, psychosexual; QoL, quality of life.

Transparent and complete information is crucial for helping patients cope throughout their lives, and patients often fully understand the information only after several appointments with health-care professionals^12,26^. Diagnostic and treatment-related information should be given in the context of typical sex differentiation and/or development for improved conceptual grasp of naturally occurring variations. The benefits and risks of eventual interventions should be discussed with the patient and their families as part of a joint process. Today, health-care providers generally agree that all children who have a chronic condition should be informed early. This agreement holds true for DSDs^35^ and is a continuous process at the child’s own pace. Explaining the condition in an age-appropriate language to the child and adolescent facilitates acceptance and can help to reduce fear and stigma. Clear and consistent communication promotes confidence in the medical team and enables future joint decision-making and therapeutic compliance^36^ (Box 3; Fig. 2). In cases where parents and/or guardians are reluctant to share developmentally appropriate and important clinical details with their children, a brief period of delay in providing this information is sometimes inevitable. At the same time, the multidisciplinary team has an important task in sensitively guiding the parents towards enhanced acceptance of the condition and reducing fears they might have around the process of informing their child.

Fertility issues are often sensitive to communicate. Nevertheless, informing individuals before adolescence in a timely and progressive manner is considered appropriate^37^. Although the possibility of transmitting a genetically determined condition can be a major concern, and germ cells are absent or severely reduced in number in the gonadal tissue of most individuals with a DSD condition, some options, such as early tissue cryopreservation, are available for a limited number of patients^38^. Although not yet clinically available, in vitro maturation of immature sperm or oocytes and harvesting germ cells from induced pluripotent stem cells are promising future techniques^39^. Therefore, stepwise decision-making in relation to gonadal surgery can guide families through this difficult process. Possibilities for fertility preservation using experimental procedures, with their ethical and economical drawbacks, should be carefully balanced against germ cell cancer risk or unwanted hormone production and considered alongside the promotion of other ways of having a family, such as adoption^40^.

Box 3 Essential components of psychosocial care to affected families **Basic requirements for dealing with patients**

- Acknowledge variety, complexity and individuality
- Create an atmosphere of appreciation and acceptance
- Provide time, empowerment and encouragement

**Information for parents**

- Biological: explain the condition as a naturally occurring variation
- Medical: explain sex and gender as non-binary concepts and in the context of sex determination and differentiation; provide precise information on the specific condition; provide information on vital, functional and elective medical interventions, including risks and benefits; and offer alternatives (for example, guidance on how to tackle potentially difficult situations and on how to raise resilient children who have a genital difference)
- Gender-related: discriminate between childhood (play) behaviour and adult gender identity; explain that the initial decisions on social gender role may be subject to later change according to the self-expression of the developing child; and put forward gender contentedness as the ultimate goal
- Use sensitive and respectful language (for example, avoid using terms such as malformation and disorder)
- Communication: listen, repeat information and ask for questions
- Promote contact with support groups and participation of appropriately trained peers in the decision-making process or in the multidisciplinary team

**Psychological counselling**

- Discuss communication within the family and social environment and support decision-making on (provisional) gender role, medical interventions and judicial options or regulations (for example, birth certificate entry)
- Promote acceptance of individual development
- Avoid emotionally driven decisions, delay non-urgent decisions (such as those on surgery) until psychological counselling has been given and promote participation of trained peers in the decision-making process

### Transition of care

Much of the literature pertaining to the medical care of individuals with a DSD comes from paediatric practice and the adult perspective is not commonly represented. Children often transition from specialized paediatric care to general adult or primary care services with potential loss of confidence in provision of DSD-specific medical management. From there, it is sometimes difficult to find a way back to an informed clinical service, especially because adult specialists who have a detailed and in-depth training in DSDs are rare. As a result, many adult patients describe difficulties finding good endocrine care^41^.

Ideally, the adult specialist that a patient is being transferred to would be involved early in the transition process. To build a trustful relationship, an open discussion of all available relevant medical data, including progressive information on any hitherto insufficiently communicated aspects of the condition, is crucial. Evolving gender identity and sexual orientation might also become important themes in the transition to adulthood. Experts in the field have developed a DSD-specific transition checklist for service users and multidisciplinary teams^27,42^ (Fig. 2).

### Multidisciplinary care in adulthood

Multidisciplinary care is equally important in adulthood as it is in childhood and can involve specialists from an even wider range of disciplines. Individuals with congenital adrenal hyperplasia (CAH) require lifelong glucocorticoid treatment and usually additional mineralocorticoid replacement^43^. Sex hormone replacement therapy (HRT) can be given to adults with non-functional or partially functioning gonads to promote cardiovascular and bone health as well as general well-being. In the absence of DSD-specific guidelines, clinical practice usually follows published recommendations that have been developed for individuals with Turner or Klinefelter syndrome^18,44^.

Fertility is markedly reduced in almost all forms of DSDs. In CAH, optimal hormonal control is required in women who seek pregnancy and in men who have testicular adrenal rest tumours^45^. Assisted reproductive techniques can offer fertility prospects to some individuals with a DSD. Microdissection testicular sperm extraction (micro-TESE) could be an option for some men who have no sperm in their ejaculate, provided that sperm is present in the testis biopsy^46^. In 2015, the first live births after uterus transplantation in women with Mayer–Rokitansky–Küster–Hauser (MRKH) syndrome were reported^47^. In women with a 46,XY DSD who have a uterus, successful pregnancies after egg donation have been described, but numbers are too low to allow conclusions regarding indications and success rates^48,49^. Careful and sensitive counselling about fertility chances as well as discussing valuable alternatives, such as adoption, are paramount. Management of vaginal hypoplasia is complex and multifaceted. Apart from a central role for the team psychologist, treating vaginal hypoplasia requires expertise with several treatment options^50^.

As a result of uncertainty regarding the genital aspect and/or function, and possibly impaired body image, many individuals who have a DSD fear intimacy and report anxiety and distress related to sexuality, resulting in a tendency to delay or avoid sexual experience^51^. The gynaecologist and urologist, along with the team’s sexologist or psychologist, are well placed to discuss such issues. Psychological support and counselling can also be offered to adults with a DSD at one or more occasions in life. Topics include dealing with the disclosure of diagnostic results or the condition itself, attaining medical files and, in patients who have not been previously or fully informed of the DSD, learning one’s story. In psychotherapy, the loss of body integrity, sexual fear and anger towards parents and the medical system are frequent concerns^52^. Importantly, engaging with (online) support groups, individual peer support and forging partnerships between communities, professionals and voluntary groups have proved beneficial for adolescents and adults living with DSDs and should be encouraged whenever possible^53^.

### Hurdles in practical implementation

With respect to the recommended provision of care by specialized multidisciplinary teams, logistical practicalities have meant that many individuals with a DSD continue to be followed by single specialists ((paediatric) endocrinologists, urologists or gynaecologists). Implementation of multidisciplinary-team-related guidelines requires drastic centralization and structural collaboration of reference centres in large networks, such as the recently established ERNs. The aims of such guidelines should prioritize the development of, and adherence to, standard care protocols and data collection while investing in sustainable registries that promote secure exchange and analysis of clinical data according to FAIR (findable, accessible, interoperable and reusable) principles^54^. Shared diagnostic strategies, research expertise and biobanks within the network can overcome differences in health-care levels. Patient engagement, at the level of both individual health-care providers and networks, is paramount. In some centres, peer counselling is a fundamental component of care before critical decision-making (for example, related to genital surgery). Others have, in collaboration with parents and representatives, developed web-based decision-support tools^55^. There are examples of support groups investing in the professional training of family members willing to provide peer support^30^. Indeed, from our experience, this training is indispensable as it enables the peer to provide non-judgemental support to the patients and it informs families in a way that encourages them to make their individual decisions.

Within the Endo-ERN, patients are represented at all levels. Patients are actively involved in network governance and development and/or the critical appraisal of network-related protocols and identified research priorities. Although such developments have been strongly encouraged by the European Commission’s Action Plan for Rare Diseases, they await further legislative and financial support from governmental bodies along with a definitive switch to a patient-centred care paradigm. Further development of peer support will require major investments in training programmes and remuneration of provided time and expertise.

## Suggested data collection across ages

### Assessment of genital status and follow-up

Impaired body experiences, such as decreased body satisfaction or decreased feeling of attractiveness in adults with a DSD, have resulted, in part, from past negative experiences^12^. Therefore, all genital assessments should be limited to a minimum and performed with great caution and with explicit consent from patients and parents and or/guardians. Repeated genital exams, medical photography without appropriate informed consent and the presence of multiple health professionals during physical examination should be strictly avoided. Recommendations on how to perform necessary genital exams in children who have genital differences, and for preparing the child for this procedure, have been described elsewhere^56^.

#### Genital assessment in neonates, children and adolescents

In male neonates and infants, description of external genitalia is facilitated by the use of a specifically designed, quantitative scoring system, the external masculinization score (EMS)^57^. A modified, non-binary version, applicable in both boys and girls and which aims to be more objective than the widely used Prader score^58^, is currently being validated in a European multicentre study. The anogenital distance correlates with prenatal androgen exposure, but standardization of this measurement is yet to be established^59^. Prepubertal girls, in general, do not require assessment of the vaginal status, especially in the absence of previous surgery. The indication and timing of such a procedure should be individualized but are usually not indicated before (induction of) puberty. When a uterus is present, a gynaecological examination should determine whether anatomy allows trouble-free menstrual flow^41^. When the measurement of vaginal length and/or vaginal examination in a pubertal girl without a uterus is planned (a procedure that is conducted mainly for deciding whether to use vaginal dilation therapy and then supporting the therapy), the need for local or general anaesthesia should be discussed and agreed upon with the individual. Reference values for genital dimensions in adult women, but not teenage girls, have been published elsewhere^60^.

#### Long-term outcome of surgical procedures

Reconstructive surgery has always been a substantial part of DSD care and has remained so for many years seemingly without debate. However, this has changed dramatically following disquieting reports of unfavourable outcomes, including high complication and/or reoperation rates and patient dissatisfaction^61–65^. A more patient-centred approach has been adopted and was informed by public discussions^66,67^. Modern reconstructive surgery claims that it is possible to create functionally and cosmetically normal-appearing genitalia; however, there is still no consensus regarding indication, timing or procedures of choice^20,68^.

The lack of consensus is partially driven by a dearth of relevant, systematic data, which is due to the rarity of the conditions, the heterogeneity of presentations, the loss of patients from follow-up into adulthood and the long interval between surgery and time of data collection. As a consequence, many studies report on long-term results of surgical techniques that are no longer in use^50,69^. As legal liability of genital surgery becomes increasingly important, centralization of expertise and structured assessment, audit and meticulous documentation of outcomes in prospective registries are paramount^19,21^. Such assessments must include documentation of complication rate, functional outcome (micturition (urination) and sexuality), cosmetic outcome, quality of life, psychosexual functioning and, finally, re-evaluation of the indication. Although genital surgery can involve a radical approach to the urinary tract, the effects on urinary function and the pelvic floor (including safe urine storage and drainage, urinary continence and risk of infection) are often insufficiently addressed^70^. Validated tools are available for clinical assessment and self-assessment of masculinizing surgery outcomes, including HOPE, HOSE, PPS and SAGAS-M^71–74^. Reliable tools for assessing outcomes of feminizing surgery are needed as today clinicians must heavily rely on their personal experience^75^.

#### Germ cell cancers

**Fig. 3:**
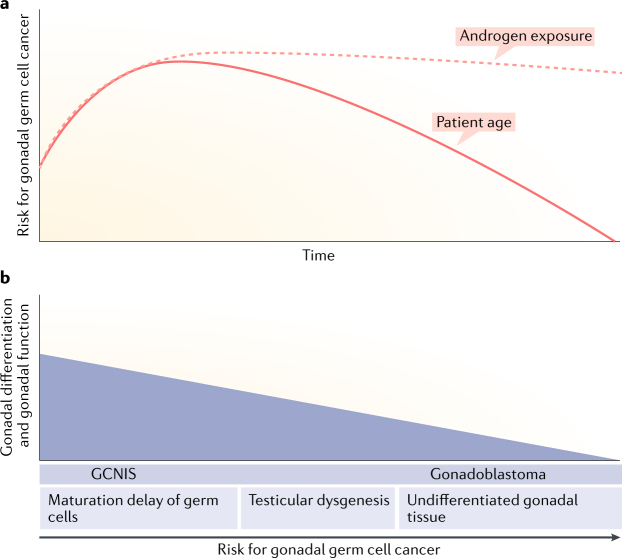
Factors influencing germ cell cancer risk in individuals with 46,XY or 45,X/46,XY DSD. **a |** Testicular germ cell cancers in the general male population have a peak incidence in late adolescence and early adulthood, as represented by the incidence projection curve (solid pink line), possibly related to the rise in androgen exposure from puberty onwards (pink dashed line, representing this hypothetical relation); this age distribution seems to be similar in individuals who have a difference of sex development (DSD). **b |** The risk of gonadal germ cell cancer in individuals with a 46,XY or 45,X/46,XY DSD is related to the degree of differentiation (or ‘testicularization’) of the gonads, which can be derived from histopathological and immunohistochemical characteristics, such as the overall morphological aspect, maturational disturbances of the germ cells, inappropriate presence of pluripotent germ cells and impaired Sertoli or granulosa cell differentiation. Germ cell neoplasia in situ is the expected precursor lesion in gonads with testicular differentiation; gonadoblastoma is typically observed in highly undifferentiated gonads. The risk is believed to be greater in abdominal than in inguinal or scrotal gonads. Genetic predisposition (for example, by the combined presence of testicular germ cell cancer-related single-nucleotide polymorphisms) can further modify the risk of gonadal germ cell cancer^78,83^. GCNIS, germ cell neoplasia in situ.

The various forms of XY and X/XY DSD have a variable risk of developing a gonadal germ cell cancer, which is further modulated by patient age, location of the gonad and, possibly, genetic predisposition^76^ (Fig. 3a). The risk is much higher in men and women with gonadal dysgenesis, especially forms that arise from early gonadal differentiation defects, such as mutations in *SRY* and *WT1*, than in individuals with ovotesticular DSD or with hormone synthesis or action disorders (for example, androgen insensitivity syndrome)^77–81^ (Fig. 3b). The underlying pathogenic mechanisms and proposed management were reviewed elsewhere in 2015 and 2017 (refs^76,82^). An increasing number of adults with a DSD have retained gonads^1^. Only in the coming decades, and with meticulous documentation of all factors involved, will the effects of advancing age, concomitant diseases, inadequate HRT, use of other medications or long-term exposure to environmental disruptors and lifestyle habits on the development and prognosis of gonadal cancer become evident. While awaiting these new data, and in the absence of reliable tumour markers or imaging technologies for early detection of precursor lesions, it seems prudent to consider gonadal biopsies to exclude the presence of germ cell neoplasia in situ or gonadoblastoma in most individuals with 46,XY or 45,X/46,XY DSD with retained gonads. Given that the age of distribution for testicular germ cell cancers is well established, such biopsies are best performed in late adolescence^82,83^.

### Somatic assessment

**Fig. 4:**
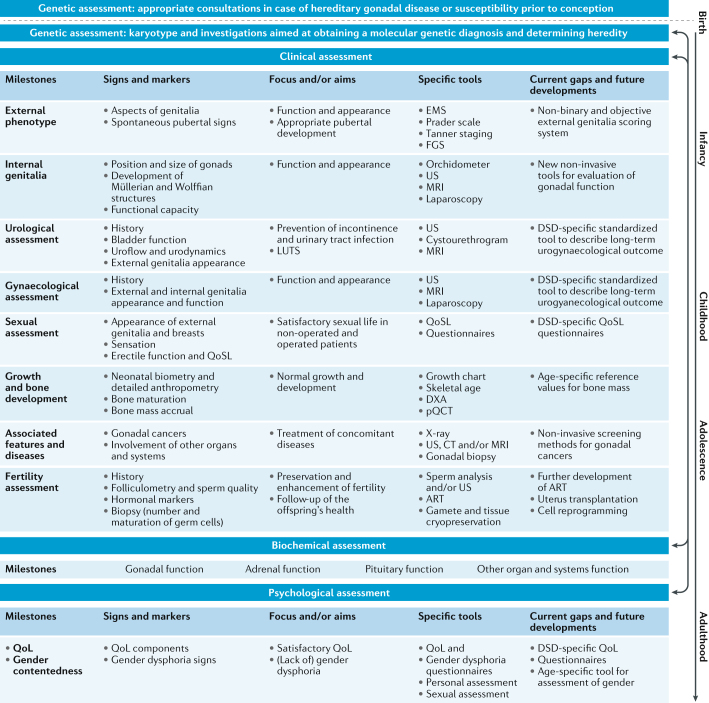
Assessment of a patient with a DSD in the clinical practice. Assessment of an individual with a (potential) difference of sex development (DSD) starts prenatally, followed by age-dependent relevant assessments throughout life. Holistic assessment includes genetic, clinical, biochemical and psychological investigations in a wide range of specific domains (left column). For each of these domains, defined signs and markers are available that can be used to screen for potential problems. The use of a pre-identified set of tools enables standardized assessment for each domain, focusing on relevant outcomes. Current gaps and future developments are listed in the right column. Dotted lines indicate inter-relations between the DSD patient and the family members and/or partners; solid lines indicate inter-relations between the different aspects of assessments according to the age of the DSD patient. ART, assisted reproductive technology; DXA, dual-energy X-ray absorptiometry; EMS, external masculinization score, FGS, Ferriman-Gallwey score; LUTS, lower urinary tract symptoms; pQCT, peripheral quantitative CT; QoL, quality of life; QoSL, quality of sexual life; US, ultrasonography.

The developmental origin of DSD conditions and the effect they can have on the functioning of several organ systems warrant extensive somatic assessment of all individuals presenting with a DSD, irrespective of age (Fig. 4). A detailed physical examination should be aimed at excluding anomalies of other organ systems, such as heart, lungs, kidneys or the skeleton, and of syndromic features, such as short fourth metacarpal or metatarsal in individuals who have 45,X/46,XY DSD or large uplifted earlobes as seen in children who have Mowat-Wilson syndrome^6,84^.

The standardization of somatic assessments is crucial to secure the validity of cross-centre data pooling with the aim of revealing hitherto unrecognized health consequences and outcomes. Indeed, scarce data suggest that adults who have a DSD experience more health problems than the general population^85,86^. Not surprisingly, morbidities related to deficiencies in or treatment with sex steroids or glucocorticoids are over-represented. Although most immediate effects of HRT, and their importance for general well-being and quality of life in adulthood, are well known, the beneficial effects of HRT on specific brain functions, such as cognition or emotional processing, especially in elderly persons, are only beginning to be understood^87^. Follow-up of long-term effects of different HRT regimens into adulthood is scarce but is much needed to aid healthy ageing.

#### Birthweight

Complications at conception or in early pregnancy and reduced fetal growth have long been associated with atypical male genital development^88^ and male subfertility^89^. Cryptorchidism occurs with increased frequency in preterm infants and infants born small for gestational age; the odds ratio for having hypospadias when born small for gestational age is 4.34 (ref.^90^). Conversely, in men with a clinical picture of partial androgen insensitivity syndrome (PAIS), a mutation in the androgen receptor is more likely if birthweight is within the normal range than if birthweight is outside the normal range^91^. Presently, it is unclear whether intrauterine growth restriction contributes to the development of hypospadias or whether both conditions result from a common underlying aetiology, but androgens per se do not seem to play a notable role in prenatal body growth. Possibly other, so-far unidentified, factors regulate differentiation and development of the urethra as well as prenatal growth^91,92^.

#### Associated congenital anomalies

Some forms of DSDs are intrinsically associated with developmental defects affecting other organ systems, reflecting the importance of many DSD-related genes, mostly those that encode transcription factors, in extragonadal embryonic development. Congenital heart defects are seen in individuals with 46,XY DSD who have *GATA4* and *FOG2* (also known as *ZFPM2*) mutations^93,94^. Cardiovascular pathology in men and women with 45,X/46,XY DSD is similar to that in women with Turner syndrome, irrespective of the clinical phenotype^95^. Coding and structural variations in *SOX9* or some of its regulatory regions can cause, apart from XX or XY DSD, a severe skeletal condition known as campomelic dysplasia^32^. Skeletal, as well as renal, involvement is common in MRKH syndrome^96^. The Denys–Drash, Frasier and the Wilms tumour, aniridia, genitourinary malformations, mental retardation (WAGR) syndromes, all three of which are caused by mutations and structural variations in *WT1*, are associated with severe kidney disorders and end-stage renal failure^97,98^. Upper urinary tract and kidney abnormalities are sporadically reported in hypospadias and androgen insensitivity syndrome^6^; however, the long-term outcome of these conditions with regard to kidney function is largely unknown.

Mutations in *SF1* (also known as *NR5A1*) were first identified in individuals with 46,XY DSD with combined adrenal and gonadal defects^99^ but have subsequently been observed in individuals with XX or XY karyotypes with a broad range of isolated gonadal or reproductive phenotypes. We currently do not know whether adrenal function can deteriorate in these persons over time. *Sf1* has also been implicated in murine spleen development, and in 2014 and 2016, asplenia and/or hyposplenia was reported in some individuals with *SF1* mutations with undetermined functional consequences^100,101^. The *FOXL2* and *RSPO1* genes are, apart from their role in ovarian development and maintenance, involved in developmental conditions affecting the eye (such as blepharophimosis, ptosis and epicanthus inversus syndrome) and skin (such as palmoplantar hyperkeratosis and a predisposition for squamous cell carcinoma)^102,103^.

#### Childhood and adolescent growth

Children with 45,X/46,XY karyotypes are at high risk of adult short stature, similar to girls with Turner syndrome, irrespective of growth hormone therapy^104,105^. The contribution of the sex chromosomes or the postnatal gonadotropin surge to growth and final height is still largely unknown. In addition, the nature of many DSD conditions, the absence of sex steroids in late childhood and/or the timing of puberty induction can alter typical growth patterns^106^. Therefore, anthropometric data supply useful information in the assessment of individuals of all ages with a DSD.

#### Bone mineralization and osteoporosis

To date, there has not been a systematic documentation of bone development in children and adolescents who have a DSD; however, decreased BMD is a common feature in adults with a DSD. Important factors that determine outcome with regard to bone strength are a late diagnosis, the type and severity of hormone deficiencies, the duration and doses of HRT, vitamin D status, physical activity, concomitant diseases, other medication and/or medications and heredity^107^.

Women with 46,XX disorders of ovarian development or maintenance have a marked decrease in BMD of the lumbar spine and femoral neck^108^. Decreased BMD has also been reported in up to 70% of women with CAH aged 30 years or older^86,109^. In this condition, compromised bone health is related not to the disease per se but to lifelong corticosteroid replacement therapy^110^. Only a handful of small-scale studies have investigated bone health in individuals with 46,XY DSD. Women with the complete form of androgen insensitivity syndrome (CAIS) who had gonadectomy have a notable decrease in BMD in comparison with male and female reference values, whereas BMD seems to be less compromised in women with CAIS who have retained testes^111,112^. Both the lack of androgenic effects at the bone level and subnormal oestrogen levels contribute to reduced bone health^110,113^. Partially virilized women with 46,XY gonadal dysgenesis have a normal BMD^114^. No reports on fracture risk in elderly individuals who have a DSD currently exist.

#### Obesity, diabetes, hypertension and cardiovascular diseases

Individuals who have a sex chromosome DSD are at increased risk of autoimmune disorders (such as type 1 diabetes mellitus and autoimmune thyroid disease), metabolic disturbances and type 2 diabetes mellitus^115–117^. The prevalence of these conditions in men and women with 46,XY DSD is poorly documented. Increased prevalence of obesity, insulin resistance and lipid abnormalities has been reported in women with CAIS, possibly due to loss of androgen receptor signalling^118^. Although genetically engineered *Sf1*-knockout mice (*Sf1*^−/−^) have increased vulnerability to develop obesity^119^, current clinical data do not support an association between *SF1* mutations and obesity in humans^120^.

Metabolic disturbances are expected in women with 46,XX DSD who have a virilizing condition. Treatment-naive and corticosteroid-treated adult women with CAH have increased BMI, insulin resistance and higher blood levels of glucose following an oral glucose tolerance test than healthy controls^121^. High glucocorticoid doses correlate positively with obesity in CAH adults^86,109^. The prevalence of the metabolic syndrome is not well defined in other DSD groups. Of note, individuals who receive HRT might be vulnerable to metabolic disturbances, and clinicians should screen these patients accordingly. Obesity can contribute to the development of hypertension and cardiovascular diseases^122^. Increased blood pressure is an intrinsic feature of some forms of CAH, such as 11β-hydroxylase deficiency, and in 17α-hydroxylase/17,20-lyase deficiency^123^. Increased blood pressure can also result from fludrocortisone overtreatment^124,125^. Whether cardiovascular function is altered in other forms of DSDs is unclear, but detailed assessment and follow-up of the cardiovascular system seem important in many individuals with DSDs, especially those on HRT.

#### Central nervous system involvement

An increased prevalence of cognitive and motor disturbances and neuropsychiatric conditions has been described in individuals with sex chromosome aneuploidies and, to a lesser extent, in individuals who have other forms of sex chromosomal DSD^126,127^. The neural system remains largely unexplored in non-chromosomal DSD. Several cases of 46,XY DSD have been associated with sensorimotor neuropathy, suggesting a heterogeneous molecular genetic basis for the neurological complications^128^. The X-linked α-thalassemia mental retardation (ATRX) syndrome is associated with genital abnormalities ranging from cryptorchidism to streak gonads and female-looking external genitalia in up to 80% of affected individuals who have a 46,XY karyotype^129^. Mental retardation is an important feature of the WAGR syndrome but not the Denys–Drash or Frasier syndromes^130^.

Psychiatric morbidity, anxiety and substance abuse are common among both women and men with CAH^131,132^. Glucocorticoid abnormalities can be responsible for reduced working memory in both girls and boys with CAH^133^, whereas prenatal exposure to excess androgens in females is probably related to alterations in aggression, activity level and gender-related interests^134^. The influence of prenatal dexamethasone treatment on the fetal brain, when used to avoid intrauterine virilization of external genitalia in pregnancies at risk of CAH, is poorly understood. Research indicates negative effects on working memory, even after short-term exposure limited to the first trimester, in unaffected individuals^135–137^. Long-term outcome data are needed to address the controversies around this treatment.

### Hormonal and genetic data

Clinical outcome is intrinsically linked to endocrine function and underlying genetic mechanisms. As illustrated in Fig. 4 and Supplementary Table 1, detailed hormonal data and molecular genetic investigations need to be meticulously collated and archived for future large-scale data analysis.

### Assessment of psychological outcome

#### Psychological adjustment and psychopathology

Some studies report increased prevalence of psychological distress, self-harm and suicidal tendencies in individuals who have a DSD^52^. Known causes include experiencing taboo, shame or secrecy; however, living with chronic illness and DSD-related physiological sequelae might also contribute to increased distress^35,138^. Peer support has been shown to have positive effects on psychological well-being^30^. Factors related to medical care, such as hormonal status and level of information sharing, can hinder or facilitate acceptance of the condition^12,26^. Documentation of the communication process will ultimately enable study of the effect of adequate (and inadequate) information and participation in decision-making on psychological adjustment and coping.

#### Gender contentedness

Assessment of gender identity and gender-related behaviour in individuals with a DSD has become a particularly sensitive topic given evolving sociological perspectives on a historically binary framework of sex and gender. Societal acceptance of non-stereotypic gender presentation and non-binary gender identities has increased in the past decade^139^. In a clinical setting, this has translated into recognition of the need for nuance in assessing gender-related presentation. For example, gender role behaviour and/or presentation should be considered as distinct from core gender identity, which is defined as the basic sense of self as male and/or female and/or other, in drawing clinical conclusions about gender development^140^. In the most recent version of the *Diagnostic and Statistical Manual for Mental Disorders* (DSM-5)^141^, the diagnostic criteria for gender dysphoria have changed to reflect this distinction. In contrast to past conceptualizations, in which a gender non-conforming presentation could have been diagnosed (or misdiagnosed) as gender dysphoria, a strong desire to be another or other gender must now be clearly demonstrated by an individual. In addition, the new formulation includes the provision for a diagnosis in individuals with a DSD, in contrast to previous editions where the presence of a DSD was considered an exclusionary criterion.

According to the literature, the above distinction is critical for assessing gender-related development in DSDs. Although variance in gender role behaviour or sexual orientation might draw clinical attention and/or warrant provision for added psychological support, distress related to core gender identity is a more serious matter, with the potential need for medical intervention, such as a revised hormone replacement regime or surgical gender reassignment^139^. Although there is a growing recognition of gender and gender identity as a continuum rather than a binary concept, the majority of individuals with DSDs identify with the gender designated at birth, that being either male or female, as do >99% of the general population^142^. However, rates of gender dysphoria, intersex, intergender identities and/or gender change are higher in patients with DSDs than in the general population^143–151^. The greater degree of variance in gender-related development in DSDs than in non-DSD populations is probably due to a complex interaction of genes, varied exposure to sex hormones in the perinatal period and again at puberty and the psychological and/or emotional sequelae of living with a DSD.

A holistic and patient-centred model of health care should be sensitive to the potential for secondary distress arising from discomfort with one’s gender. Therefore, enquiries about an individual’s gender experience and gender-related contentedness are important. At the same time, the assessment should be framed in a way that does not stigmatize or pathologize any particular gendered presentation. Questions can be asked in an open-ended manner so as not to raise a ‘typical or atypical’ dichotomy. In addition, the criterion of note with respect to potential clinical concern is the presence of distress. ‘Atypical’ presentation is very often not accompanied by distress, and gender-related contentedness is mostly satisfactory in such cases^150^. The aim of these questions is to give patients and families support relevant to physical, psychological and emotional aspects of DSDs in an open-minded and caring environment^150^. A review of psychological assessment across the lifespan addresses the nuance and complexities of gender development in DSDs^152^.

### Quality of life

Optimizing quality of life is a primary goal of holistic care and outcome-focused research. Given the rarity of the individual conditions, most studies include a conglomerate of diagnoses, which yields inconsistent results and the general conclusion that DSD-related quality of life is understudied^153,154^. In addition, earlier studies report on women who have not experienced the current patient-centred standard of care by a multidisciplinary team. Studies on larger series of mainly XY DSD conditions show overall equal quality of life, compared with the local general population, although some studies suggest suboptimal quality of life, specifically in the social and psychological, but not physical, domains^154–158^. In conclusion, appropriate questionnaires should be designed for assessing quality of life in individuals with diverse sex development, focusing on partnership, sexuality and fertility, in addition to those assessing physical quality of life.

## Conclusions

Within the management of DSDs, many unsolved questions remain, especially in older age groups. The establishment of proper genotype–phenotype correlations and addressing the identified gaps in our current knowledge are primary tasks of future research. To reach these goals, it is crucial that patient follow-up continues throughout their lives in dedicated reference centres, where possible. Prospective multicentre data collection in adults is one of the most urgent needs given that this has been a long-neglected group with respect to clinical research.

According to a scarce and dispersed literature, many adults who have a DSD are probably at increased risk of various cardiovascular, metabolic and neuropsychological comorbidities, possibly related to the specific genetic constitution or to current treatments. The benefits and risks of HRT in patients with DSDs specifically are largely unknown and need further studies.

Concerns exist with regards to the effects of delayed genital and gonadal surgery on social acceptance, psychological well-being, parent–child bonding, body image and sexual functioning as well as the malignancy risk of retained gonads. Studies assessing the effect of deferred surgery on the above domains and comparing psychological outcomes with and without surgery are underway. In addition, insight into reasons why families might sometimes insist on having genital surgery for their child and investment in support tools and guidance for families and children living with atypical genitalia are urgently needed. Meanwhile, an appropriate balance between genital surgery on the one hand and the protection of human rights and dealing with ethical dilemmas on the other must be found. Longitudinal studies of genital surgery that focus on genital outcomes, and that neglect urinary functioning, can lead to inappropriate conclusions.

The creation of multidisciplinary teams dedicated to the management of DSD conditions worldwide poses its own challenges. Apart from economical limitations and practical hurdles, training of staff with respect to the acquisition of knowledge, as well as communication skills, is an ongoing endeavour^159^. Examples that outline the role of team members as well as team responsibilities are available^28,160^. Developing networks of peers who have received specific training and who can be included in the multidisciplinary team can enrich the decision-making process with a (so far) under-represented and much broader perspective.

Towards the aims of facilitating systematic and structured patient follow-up in parallel with accumulating much-needed data for future DSD research, consensus among health-care providers, researchers and service users is crucial. To avoid selection bias in patient-reported outcomes, we recommend a standardized, non-binary and holistic assessment of individuals at specific life stages and in the context of mandatory clinical assessment or review. Prospective studies are best managed in a multidisciplinary setting, including both paediatric and adult specialists, with the aim of systematic, longitudinal data collection using evidence-based, standardized assessment tools and protocols. By doing so, critical developmental milestones and/or long-term sequelae associated with various DSD conditions can be captured. Rapid translation of obtained clinical research data into evidence-based practice requires investment in enhanced communication strategies, systems for electronic data storage, exchange and analysis, fostering a long-term vision of the organization of health-care structures and improved professional and public understanding of the needs and actions that drive progress on this matter. Large-scale networks that are currently being formed, such as Endo-ERN in Europe and the DSD Translational Research Network in the United States, are promising developments in this direction. At the root of the endeavour, holistic health care and clinical research should always be performed in accordance with each patient’s expectations and with sincere respect for their integrity.

## Electronic supplementary material

Supplementary Information
